# The mechanism of silicon on alleviating cadmium toxicity in plants: A review

**DOI:** 10.3389/fpls.2023.1141138

**Published:** 2023-03-23

**Authors:** Lei Hou, Shengzhe Ji, Yao Zhang, Xiuzhe Wu, Li Zhang, Peng Liu

**Affiliations:** College of Plant Protection, Shandong Agricultural University, Taian, Shandong, China

**Keywords:** antioxidant, cadmium accumulation, cadmium toxicity, silicon, water balance

## Abstract

Cadmium is one of the most toxic heavy metal elements that seriously threaten food safety and agricultural production worldwide. Because of its high solubility, cadmium can easily enter plants, inhibiting plant growth and reducing crop yield. Therefore, finding a way to alleviate the inhibitory effects of cadmium on plant growth is critical. Silicon, the second most abundant element in the Earth’s crust, has been widely reported to promote plant growth and alleviate cadmium toxicity. This review summarizes the recent progress made to elucidate how silicon mitigates cadmium toxicity in plants. We describe the role of silicon in reducing cadmium uptake and transport, improving plant mineral nutrient supply, regulating antioxidant systems and optimizing plant architecture. We also summarize in detail the regulation of plant water balance by silicon, and the role of this phenomenon in enhancing plant resistance to cadmium toxicity. An in-depth analysis of literature has been conducted to identify the current problems related to cadmium toxicity and to propose future research directions.

## 1 Introduction

Cadmium is a nonessential, highly toxic, heavy metal element and a widespread environmental pollutant that is persistent, nonbiodegradable and bioaccumulative even at low concentrations (Hussain et al., 2019). Upon entering the food chain, cadmium seriously threatens the health of animals and humans (Krämer and Chardonnens, 2001; Fulekar et al., 2009; Liu et al., 2019). Contamination of soil with cadmium severely affects plant growth resulting in leaf yellowing, stunted growth, reduced yield, reduced enzyme activity, reactive oxygen species (ROS) accumulation and protein denaturation (Bovetl et al., 2003; Fan et al., 2010; Gallego et al., 2012; Rizwan et al., 2017; Rizwan et al., 2018; Hamid et al., 2019). Owing to its high mobility in soil-plant systems, cadmium is easily absorbed by plants. Therefore, a reliable strategy to reduce cadmium toxicity and minimize its accumulation in plants is urgently needed to improve plant growth and ensure food safety.

After oxygen (47%), silicon is the second most abundant element (27.7%) in the Earth’s crust. Although silicon is not essential for the survival of terrestrial higher plants, it plays a variety of roles to ehhance plant growth, especially under biotic and abiotic stress conditions (Epstein, 1994; Luyckx et al., 2017). Silicon can improve soil properties, such as enhancing soil microbial communities, regulating nutrient cycling, enhancing soil water retention and providing a more favorable growing environment for plants (Bhardwaj et al., 2022). Silicon is abundant in nature and often exists in inert forms that are inaccessible to plants. In contrast, soluble, bioavailable monosilicic/protosilicic acid [Si(OH)_4_/H_4_SiO_4_] is typically present in soil at concentrations of (0.1–0.6 mM) (Epstein, 1994). Plants absorb silicon from the soil exclusively in the form of H_4_SiO_4_, which is the only plant-available form of silicon in the soil. After absorption, H_4_SiO_4_ is transported radially into the root cortex *via* the cytoplasm or plasmodesmata (Hodson and Sangster, 1989; Ma et al., 2006). Once H_4_SiO_4_ is loaded into the xylem, it is rapidly translocated to the aboveground plant parts through the transpiration stream (Lux et al., 2020). In organs such as stems and leaves, H_4_SiO_4_ is unloaded from the xylem into the extracellular apoplastic space. Subsequently, through transpiration and a series of biochemical reactions, H_4_SiO_4_ forms insoluble silica molecules and is ultimately deposited as a polymer of hydrated amorphous silica (SiO_2_-nH_2_O) in the cell lumen, cell wall, intercellular space or between the epidermal cell wall and the cuticle (Hodson and Sangster, 1989; Ma et al., 2006).

Silicon has been shown to benefit plant growth and reduce the toxic effects of cadmium (Ma and Yamaji, 2008; Gao et al., 2018; Hajiboland et al., 2018; Kollárová et al., 2019; Gheshlaghpour et al., 2021). In this review, recent research on the mechanism of silicon-induced mitigation of cadmium toxicity in plants is summarized. To date, studies on the mechanism of silicon-mediated mitigation of cadmium toxicity have mainly focused on reducing cadmium uptake and transport, increasing antioxidant properties, improving photosynthesis, promoting nutrient uptake and maintaining the integrity of the cell structure. This review highlights the novel hypothesis that silicon alleviates cadmium-induced damage to plants through the ability to alter water relations to dilute the cadmium concentration in plant tissues.

## 2 Silicon enhances plant growth under cadmium toxicity

Cadmium accumulation can severely inhibit plant growth. However, plant biomass and morphological traits, such as leaf length, leaf area, root length, root volume and root tip number, are enhanced by silicon under cadmium toxicity (Farooq et al., 2013; Rahman et al., 2021c; Seyed, 2022) (**Table 1**). Disruption of photosynthesis is a major impact of cadmium toxicity, which can be alleviated by silicon, for example, in rice (Nwugo and Huerta, 2008; Li et al., 2018), cucumber (Feng et al., 2010), cotton (Farooq et al., 2013), maize (Vaculík et al., 2015), wheat (Sun et al., 2016; Shi et al., 2018) and lupin (Sun et al., 2016). Chloroplasts are organelles that perform photosynthesis, and their ultrastructure plays a vital role in this process. With the accumulation of cadmium in tissues, the concentration of ROS increases, and the cell membrane system is damaged, especially when the chloroplast membrane is damaged, which will inhibit photosynthesis (Feng et al., 2010; Pereira AS et al., 2018). Silicon application has been reported to alleviate cadmium-induced cell ultrastructure damage in a variety of plant species (Zhao et al., 2022).

**Table 1:** Effect of silicon on plant growth and development to plants under cadmium poisoning.

Species	Silicon concentration	Cadmium concentration	Effect	Reference
Pea	100, 200, 300 ppm Si	50, 100 mg kg^−1^ CdCl_2_	The plant height↑by 37.45% and 36.26%, leaf area↑by 33.99% and 31.87%, dry weight of shoot↑by 31.48% and 37.16, and root dry weight↑by 36.49% and 29.97%, at 300 ppm exogenous Si. The levels of chlorophylls (a and b)↑.	(El-Okkiah et al., 2022)
Wheat	25, 50, 100 mg kg^-1^ SiNPs	7.67 mg kg^-1^ Cd	The plant height↑by 5.0%, 17.6%, 25.2%, at 25, 50, 100 mg kg^-1^ Si. Chlorophyll a content↑by 17.2%, 28.5%, 44.3%, at 25, 50, 100 mg kg^-1^ Si. Chlorophyll b contents↑by 21.6%, 43.5%, 65.6%, at 25, 50, 100 mg kg^-1^ Si.	(Khan et al., 2021)
Rice	1, 2, 4, 6 g kg^−1^ Na_2_SiO_3_·9H_2_O	0.53, 3.51 mg kg^−1^ Cd	Addition of 4 g kg^-1^ Si increased the net photosynthetic rate (27%; 45%) of cadmium-poisoned (0.53; 3.51 mg kg^−1^) plants.	(Cipriano et al., 2021)
Pepper	2 mM Na_2_SiO_3_	0.1 mM CdCl_2_	The shoot, root and total plant dry mass↑by 55.3%, 31.6% and 50.3%, at 2.0 mM Si. The chlorophyll a and b contents and efficiency of photosystem II content↑by 32.6%, 23.0% and 26.4%, at 2.0 mM Si.	(Kaya et al., 2020)
Rice	1.5 mM NaSiO_3_·9H_2_O	100 μM CdCl_2_	The aboveground biomass↑, underground biomass↑, shoot and root lengths↑, the chlorophyll content↑by 9.7%.	(Chen et al., 2019)
Wheat	300, 600, 900, 1200 mg L^-1^ SiNPs	0.93 mg kg^−1^ Cd	The shoot length and grain weight↑by 14, 30, 43, 51%, at 300, 600, 900, and 1200 mg L^-1^ Si. The grain weight↑by 24%, 43%, 52%, 62%, at 300, 600, 900, and 1200 mg L^-1^ Si. The shoot dry weight, root dry weight, shoot length, grain weight, spike length, spike dry weight↑by 66, 67%, 51%, 62%, 53%, 66%, at 1200 mg L^-1^ Si. The photosynthetic rate↑by 79%, at 1200 mg L^-1^ Si.	(Hussain et al., 2019)
Rice	0.03% w/w K_2_SiO_3_	0.52 mg kg^−1^ Cd	The shoot and root dry weights↑. The photosynthetic rate↑by 83.9% with spliting application of Si at three growth stages (S1+S2+S3).	(Rehman et al., 2019)
Wheat	3 mM K_2_SiO_3_·nH_2_O	2 mM Cd^2+^	The Si applied as soil addition was the best treatment. The shoot length↑by 69.8%, leaf area↑by 82.7%, seedling FW↑by 86.8% and seedling DW↑by 107.7%. The net photosynthetic rate↑by 62.5%.	(Howladar et al., 2018)
Wheat	1 mM Na_2_SiO_3_·9H_2_O	5, 20 µM CdCl_2_·3/2H_2_O	Addition of Si increased the above-ground dry weight (47%) of cadmium-poisoned (5 μM) plants. Addition of Si increased the net photosynthetic rate (27%; 45%) of cadmium-poisoned (5; 20 μM) plants.	(Shi et al., 2018)
Rice	120 mg L^−1^ Na_2_SiO_3_	1, 5 mg L^−1^ Cd	Addition of Si increased the above-ground dry weight (51%;81%) of cadmium-poisoned (1; 5 mg L^−1^) plants. Applied Si can restore the morphology and structure of chloroplasts.	(Guo et al., 2018)
Wheat	0.6 mM SiO_2_·nH_2_O	15 μM CdCl_2_·H_2_O	Shoot dry matter of Sehar-2006↑by 16%. The photosynthetic rate of Sehar-2006↑by 44%.	(Naeem et al., 2018)
Rice	42 mg kg^−1^ K_2_SiO_3_·nH_2_O	50 mg kg^−1^ CdCl_2_·2.5H_2_O	Shoot dry weight↑by 28% in Feng-Hua-Zhan. Shoot dry weight↑by 48% in Hua-Hang-Si-Miao. The total chlorophyll content↑by 75%. The chlorophyll fluorescence parameters by↑73%.	(Huang et al., 2018)
Tobacco	1, 4 g kg^−1^ Na_2_SiO_3_·H_2_O	1, 5 mg kg^−1^ CdCl_2_·H_2_O	Root dry weight↑by 17% (Cd1+Si1 compared to Cd1). Stem dry weight↑by 44% (Cd5+Si1 compared to Cd5). Leaf dry weight↑by 70% (Cd5+Si4 compared to Cd5). The total biomass↑by 47% and 49% (Cd5+Si1 and Cd5+Si4, compared to Cd5). The contents of chlorophyll a, chlorophyll b, total chlorophyll, and carotenoids↑by 40.4%, 23.9%, 35.2%, and 41.1% (Cd5+Si4 to Cd5).	(Lu et al., 2018)
Gladiolus	200 mg L^-1^ Si	50 mg kg^−1^ CdSO_4_·8H_2_O	The above-ground and root dry weight↑by 22% and 11%. The photosynthesis↑.	(Zaheer et al., 2018)
Maize	5 mM Na_2_SiO_3_	5, 50 μM Cd(NO_3_)_2_·4H_2_O	Addition of Si increased above-ground dry weight (32%; 21%) of cadmium-poisoned (5; 50 μM) plants. The addition of Si increased the net photosynthetic rate of Cd-poisoned plants.	(Vaculík et al., 2015)
Cotton	1 mM Na_2_SiO_3_	1, 5 μM CdCl_2_	Addition of 1 mM Si increased root dry weight (25%; 62%), stem dry weight (26%; 35%), and leaf dry weight (31%; 57%) of cadmium-poisoned (1; 5 μM) plants. The addition of 1 mM Si increased the net photosynthetic rate (90%) of cadmium-poisoned (5 μM) plants.	(Farooq et al., 2013)

"↑"="increased".

In rice, silicon ameliorates cadmium-induced changes in chloroplast ultrastructure by ensuring the integrity of chloroplasts and membranes (Guo et al., 2018). In addition, silicon-enhanced regeneration of cell walls by maize protoplasts may also be used to maintain chloroplast structure (Kollárová et al., 2019). Cadmium negatively affects the formation of cystoids in the chloroplasts of bundle sheath cells, which is mitigated by silicon and the improved cystoid formation may contribute to enhance photosynthesis and then increase biomass (Vaculík et al., 2015). Cadmium inhibits the activity of chlorophyll synthase and increases the activity of chlorophyllase, thus decreasing chlorophyll content (Ekmekçi et al., 2008; Vaculík et al., 2015). Silicon increases the content of photosynthetic pigments in tobacco under cadmium toxicity and improves the efficiency of light energy utilization (Feng et al., 2010; Luyckx et al., 2021). Cadmium toxicity also causes changes in leaf structure, stomatal size and density, which ultimately inhibit photosynthesis (Shi and Cai, 2008). However, in the presence of silicon, stomatal density is increased and results in a high gas diffusion rate (Cipriano et al., 2021). Moreover, silicon deposition is beneficial for the plant to maintain an erect habit, especially under stress and to promote leaf positioning favorable for light interception and efficient photosynthesis (Epstein, 1994).

## 3 Silicon reduces the uptake of cadmium transport by plants

The phytotoxic effects of cadmium present in soil are not exerted until it is absorbed by plants and transported to various tissues and organs. There are two means for cadmium to enter the root: the apoplast pathway and the symplast pathway (Redjala et al., 2009; Song et al., 2017). In the apoplast pathway, cadmium present in the soil is absorbed by plants roots *via* free diffusion; however, instead of entering the cells, cadmium passes through voids in the cell walls of rhizodermis and cortex to enter the xylem and phloem (Degryse et al., 2006; Tao et al., 2019). The symplast pathway refers to the transport of cadmium through plasmodesmata from one cell (protoplast) to the other using cytoplasmic continuum; and cadmium transport across membranes, this process is mediated by transporters located in the cell wall (Kreszies et al., 2018). Silicon interferes with these two transport pathways in various ways, inhibiting the accumulation of cadmium in plants, thereby reducing its harmfulness to many plants (Kabir et al., 2016; Song et al., 2021).

### 3.1 Formation of physical barriers (inhibition of the apoplast pathway)

The formation of a physical barrier in the apoplast space reduce cadmium transport rates, thereby reducing the accumulation and distribution of cadmium in the cytoplasm (Ye et al., 2012; Zhang et al., 2014; Luyckx et al., 2017; Riaz et al., 2022). Silicon creates a physical barrier through the formation of specific cells (siliceous cells) that reduce the uptake and transport of cadmium in plants (Ma and Yamaji, 2008). Siliccon is deposited in root tissues through three main modes: (1) impregnation of the endodermal cell wall; (2) formation of silica aggregates associated with the inner tangential wall of the endodermal cells;and (3) formation of silica aggregates or phytoliths in specific cells associated with thick-walled sclerenchymatous tissues (Lux et al., 2020). After its absorption by plant roots exclusively as H_4_SiO_4_, silicon binds to cell wall components including hemicellulose, pectin, callose, cellulose and dextran to form SiO_2_ precipitates (Guerriero et al., 2016). Experiments on rice suspension cells show that hemicellulosic polysaccharides in the cell wall exhibit greater silicon-binding capacity than pectic polysaccharides (Ma et al., 2015). Silicon significantly increases the content of total polysaccharides and their components (pectin and cellulose) in the root cell wall and improves the ability of cadmium to bind to pectin and cellulose, which decreases cadmium transport efficiency through the apoplast pathway (Cai et al., 2022). The specific amino acid composition of cell-wall-localized proteins may also lead to silicification (Guerriero et al., 2016). Silicon increases the cation exchange capacity of the cell wall; cadmium binds to the cell wall to a greater extent in the presence of silicon than in its absence (Lukačová et al., 2013). Heavy deposition of silicon near the cortex, as aggregates or phytoliths, may reduce the cell wall porosity of the internal tissues of the root, especially in the inner cortex (Lux et al., 2020). Massive deposition of SiO_2_ near the endodermis physically blocks plasmodesmata flow through the root, thereby inhibiting the plasmodesmata transport of cadmium to reduce its toxic effects on plant cells (Shi et al., 2005).

In addition, the chemically heterogeneous walls resulting from silicon modification may provide additional binding sites for cadmium (Liu et al., 2013a). In rice and maize, silicon application enhances cell wall suberization and lignification and promotes Casparian strip formation (Fleck et al., 2011; Vaculík et al., 2012). In wheat plants exposed to long-term cadmium stress, silicon promoted suberin deposition in the root endodermis and decreased cadmium concentration in the apoplastic fluid of the shoot by almost 38% (Wu et al., 2019). Additionally, the ratio of root endothelium cell wall to the whole cell volume was higher in the cadmium+silicon treatment than in the cadmium-only treatment (Lukačová et al., 2013).

More than 90% of the H_4_SiO_4_ taken up by roots is transported to the aboveground organs, along with the transpiration water flow, through the xylem. In this process, silicon is also deposited in the leaf epidermal cell wall, which decreases the efficiency of cadmium transport (Lux et al., 2003; Keller et al., 2015). When cadmium reaches the leaf, silica aggregates in the cell wall, as a physical barrier, suppressing cadmium from entering the cells (Farnezi et al., 2020).

### 3.2 Downregulation of cadmium uptake and transport-related genes (inhibition of the symplast pathway)

In addition to the formation of physical barriers in the cell wall, silicon also affects the expression of genes involved in cadmium uptake and transport. The main cadmium transporter genes encode proteins belonging to the ZRT-like protein (ZIP), IRT-like protein (IRT), natural resistance-associated macrophage protein (Nramp), yellow stripe-like transporter (YSL), low affinity cation transporter (LCT), P-type adenosine triphosphatase (ATPase, HMA) and cation diffusion facilitator (CDF) families. Among these proteins, ZIPs, IRTs, Nramps, YSLs and LCTs mediate cadmium uptake from the soil by roots and its transport to aboveground plant parts, while HMAs and CDFs are involved in the efflux of cadmium to the cytoplasm or its transport into the vacuoles. *Nramp5*, a key transporter protein for cadmium uptake by the root system, is located at the plasma membrane and is highly expressed in the root maturation zone, responsible for the transport of cadmium from the outside into the root cells (Sasaki et al., 2012; Riaz et al., 2021). Silicon downregulates the expression of *Nramp5* in rice cells under cadmium toxicity (Ma et al., 2015). Treatment of rice suspension cells with silicon nanoparticles (SiNPs) reduces the expression levels of the genes encoding the cadmium transport proteins *OsLCT1* and *OsNramp5* (Cui et al., 2017). Due to the unique N-terminal sequence, the ATPase in plants is also named as HMAs. Among them, HMA3 is the main protein responsible for the transport of cadmium chelates into vesicles (Mohabubul Haque et al., 2022). The *OsHMA3* mediates the sequestration of cadmium in the root cell vacuole (Riaz et al., 2021). The transport of cadmium into vesicles may be an important mechanism for cadmium detoxification in plants (Li et al., 2021). Consistent with this, silicon upregulates the expression of *OsHMA3* in the root system (Cai et al., 2022). Cadmium toxicity increases the expression of four genes (*Nramp5*, *Nramp1*, *HMA2* and *HMA3*) that encode cadmium transporter proteins in rice. Exogenous application of silicon downregulates the expression of these four genes under cadmium stress (Chen et al., 2019). A homolog of *OsHMA3*, *OsHMA2*, localizes to the plasma membrane of root pericycle cells and is involved in the root-to-shoot translocation of cadmium; however, silicon downregulates the activity of *OsHMA2* (Shao et al., 2017). Located in the rice bast, *OsLCT1* mediates the transport of cadmium to the grain (Uraguchi et al., 2011). Silicon reduces the promotion of *OsLCT1* expression by cadmium (Greger et al., 2016). Thus, silicon application can minimize cadmium-induced damage to plants by reducing the uptake and transport of cadmium (Rizwan et al., 2012; Vaculík et al., 2012).

## 4 Coprecipitation

The coprecipitation of silicon and cadmium in soil reduces the availability of soil cadmium (Zhao et al., 2020). Silicon improves soil physical and chemical properties, reduces soil acidity (i.e., increases soil pH) and coprecipitates with heavy metals in the soil, forming metal-silicate complexes, to reduce the uptake of heavy metals by plants (Khan et al., 2021; Bhardwaj et al., 2022). Soluble silicates hydrolyze in soil solution and produce gel-like metasilicic acid, which binds heavy metals and changes the form of the metal from toxic to non-toxic (Bhat et al., 2019). Consistent with this result, the addition of SiNPs to cadmium-contaminated soil decreases the content of biologically effective cadmium in the soil (Hussain et al., 2019). Studies show that silicon-rich amendments fix copper, cadmium, and zinc in polymetallic acidic soils by increasing soil pH, and that metals are mainly in the form of silicates, phosphates, and hydroxides during amendment treatment exist (Gu et al., 2011). The use of silicon in the form of calcium silicate also changes the distribution of cadmium and zinc in soils, which are present in more stable forms, such as complexes with organic matter and crystalline iron oxide (Da Cunha et al., 2008). Among the four amendments tested on cadmium-contaminated soil, silicon showed the greatest reduction in the concentration of fast-acting cadmium (56.28%), greatly changed the soil microbial community (especially acidophilus and chiguria) and significantly reduced the bioavailability of cadmium to plants (Song et al., 2021).

**Figure 1:**
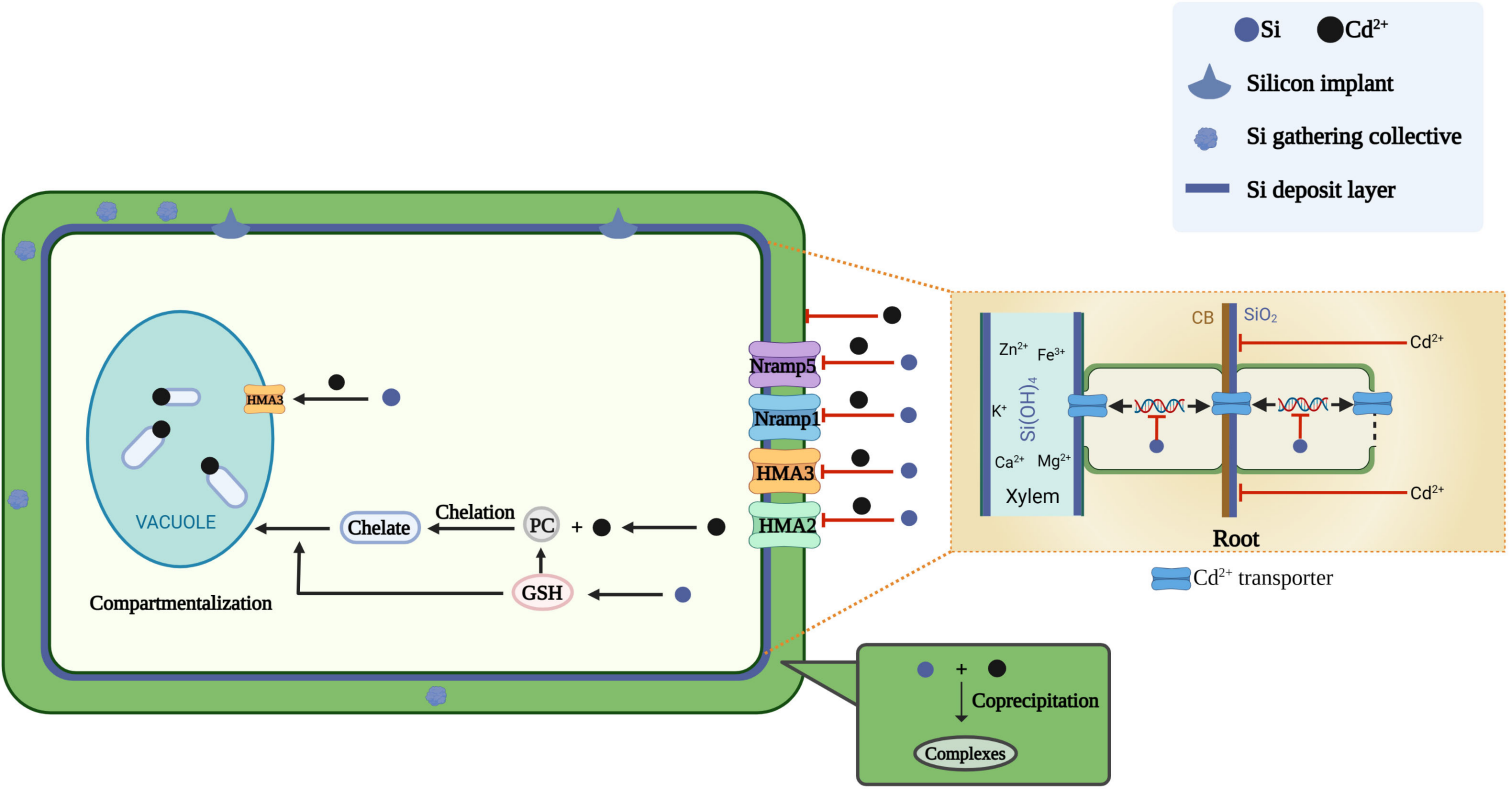
Mechanisms of silicon on reducing cadmium toxicity. The underlying mechanism includes (і) enhancing plant growth by promoting photosynthesis and nutrition uptake; (ii) reduction of cadmium accumulation due to silicon decreases cadmium uptake&transport gene expression and its deposition forms a physical barrier; (iii) the co-precipitation, compartmentalization of Cd^2+^. Cartoon pictures were created with BioRender.com.

The coprecipitation of silicon and metals in the cell walls of roots and leaves is an additional means of mitigating metal toxicity (**Figure 1**) (Wang et al., 2000; Ye et al., 2012; Cai et al., 2022). Cadmium and zinc co-precipitate with silicon in the cell walls of epidermal, ectodermal, endodermal, mesocolonial sheath and xylem cells in maize roots (Da Cunha and Do Nascimento, 2009). Similarly, the coprecipitation of silicon with cadmium in rice stems reduces the metal concentration in the leaves (Gu et al., 2011). Silicon coprecipitates with cadmium and zinc in the leaf cell cytoplasm and vesicles to form Si-metal complexes that contribute to cellular detoxification (Da Cunha and Do Nascimento, 2009). Increased silicon content in the cell wall may provide additional cadmium-binding sites (Cui et al., 2017). In the rice cell walls, electrostatic interactions between the silicon–hemicellulose matrix and cadmium cations, co-deposition of silicon with cadmium inhibit cadmium ion uptake and lead to *in vivo* detoxification of cadmium (Ma et al., 2015). This mechanism explains how silicon inhibits cadmium uptake and transport at the single-cell level. Moreover, silicon application may lead to an increase in root secretions, which can reduce heavy metal uptake by the roots because of the chelation of heavy metals (Keller et al., 2015).

## 5 Compartmentalization reaction

Vacuoles are important storage organelles for many ions, and high-level accumulation of toxic ions in vacuoles is an important strategy employed by plants to enhance heavy metal tolerance (Sterckeman and Thomine, 2020).

Phytochelatins (PCs) are a class of small biological molecules, with the structure (γ-Glu-Cys)n-Gly (n=2~11), synthesized by plant chelating peptide synthase (PCS) upon plant exposure to cadmium or other metals and oxygen-containing anions (Satofuka et al., 2001; Vestergaard et al., 2008). In plant cells, cadmium usually binds to PCs to form cadmium-PC complexes, which are then transported to and sequestered in vesicles to avoid further damage to cellular organelles (Shanmugaraj et al., 2019). Silicon increases the concentration of cadmium bound to PCs in rice (Cai et al., 2022). Similar result was also proved that the concentration of PCs with expression of *OsPCS1* in roots of cadmium-stressed was significantly induced when subjected to silicon treatment (Bari et al., 2020). PC2 and PC3 are the main PCs that synthesized in the cytoplasm of root cells after cadmium exposure (Volland et al., 2013). Silicon promotes the production of PC2 and PC3, thereby sequestering more PC-bound cadmium in the vacuole and limiting the migration of cadmium from roots to shoots (Wei et al., 2021). Glutathione both acts as a precursor for production of PCs which chelate cadmium in cells to form PC-cadmium complexes and also promotes the transport of these complexes into the vacuoles to reduce the cadmium concentration in the cytoplasm (Huang et al., 2021; Luyckx et al., 2021). Silicon increases glutathione and PC synthesis, allowing the plants to efficiently cope with oxidative stress through the improvement of cadmium sequestration on thiol groups in the roots (Luyckx et al., 2021). Stimulation of glutathione synthesis by silicon under cadmium stress was also found in rice (Farooq et al., 2016), sugar beet (Kabir et al., 2021), maize (Singh et al., 2019), wheat (Thind et al., 2020), and pepper (Kaya et al., 2020).

Heavy metals form complexes with PCs, which are transported to and sequestered in vesicles by ATP-binding cassette (ABC) transporters (Sharma et al., 2016). *OsHMA3*, a heavy metal ATPase, is a cadmium efflux protein to the vesicles and facilitates cadmium sequestration in the root vesicles (Sasaki et al., 2014). Rice under cadmium toxicity was mostly up-regulated in ABC transporter proteins after silicon treatment, which may contribute to the compartmentalization of cadmium in vesicles (Sun et al., 2022). The addition of SiNPs increases the expression of rice heavy metal ATPase 3 (*OsHMA3*) localized in the vacuole membrane and increases cadmium translocation into the vacuoles, thus reducing the effects of cadmium toxicity (Cui et al., 2017). Another heavy metal binding ligand, metallothioneins (MTs), have also been found in many plant species (Cobbett and Goldsbrough, 2002).

## 6 Silicon improves plant mineral nutrient supply

High concentrations of cadmium reduce the ability of plants to absorb and transport nutrients and disrupt mineral metabolism, leading to nutrient deficiencies (Luyckx et al., 2021). Cadmium competes with mineral nutrient ions for the same transport system, resulting in the shortage of nutrients required for plant growth and development (Alcántara et al., 1994; Sarwar et al., 2010; Nazar et al., 2012). Silicon facilitates the uptake and utilization of plant nutrients was summarized in **Table 2**. This may be one of the reasons why silicon promotes plant growth under cadmium toxicity (Pilon et al., 2013; Hernandez-Apaolaza, 2014). Exogenous application of silicon promotes the uptake of nutrients by plant roots, including mineral elements such as Zn, Fe, Mn, Ca, Mg, P, and K, thereby offsetting the shortage caused by cadmium and thus promoting plant growth (Keller et al., 2015; Wang et al., 2015; Rahman et al., 2021c). The effect of silicon on the concentration of mineral elements varies depending on the crop type, plant organism and type of mineral element. In poplar callus cells, silicon attenuates plasma membrane damage caused by cadmium toxicity and promotes the uptake and transport of nutrients including Ca, K, Mg, P, Fe, Zn, and Mn after 3 and 9 weeks of cadmium treatment (Kučerová et al., 2020). In hemp, cadmium stress increases the content of Ca, P and S in stems and leaves and that of Fe in roots but decreases the content of Fe in leaves. Additionally, silicon greatly increases Fe concentration in the leaf and alleviates cadmium-induced reduction in Ca concentration in the root and cadmium-induced accumulation of P in the shoot (Luyckx et al., 2021). On the contrary, silicon has no significant impact on S accumulation in the shoot under cadmium stress (Luyckx et al., 2021). Foliar application of silicon increases the concentration of N and P; but decreases the concentration of Na. Because silicon has no effect on the concentration of K, treatment with silicon decreases the ratio of K^+^/Na^+^ (Rady et al., 2019). In ajwain, cadmium decreases Fe, Mg, K and Ca both in shoot and root (Javed et al., 2020).

**Table 2:** Effect of silicon on the supply of mineral nutrients to plants under cadmium poisoning.

Species	Silicon concentration	Cadmium concentration	Effect	Reference
Wheat	1 mg kg^−1^ CaSiO_3_	10 mg kg^−1^ CdSO_4_·8H_2_O	Applied Si increases Zn, Fe, Cu content in roots and grain.	(Farooqi et al., 2022)
Bean	20 mg L^−1^ SiNPs	1, 1.5 and 2 mM CdCl_2_	Silicon enhancing K^+^ accumulation is beneficial to reduce stress effect.	(Koleva et al., 2022)
Hemp	2 mM H_2_SiO_3_	20 μM CdCl_2_	Cd decreased Fe and K concentration in the leaves. The addition of Si strongly increases leaf Fe content.	(Luyckx et al., 2021)
Wheat	1 and 3 mM Na_2_SiO_3_	50 and 200 µM CdCl_2_	Silicon increases the of N, P, K, Ca, Mg, and Zn.	(Rahman et al., 2021c)
Wheat	1.5 and 3 mM Na_2_SiO_3_	10 and 25 mg kg^-1^ CdCl_2_	Cadmium poisoning reduced Fe^2+^, Zn^2+^, Mg^2+^ contents in roots and shoots, and silicon application increased Fe^2+^, Zn^2+^, Mg^2+^ contents in roots and shoots.	(Thind et al., 2021a)
Ajwain	1.5 and 3 mM K_2_SiO_3_	1.5, 3 mM CdCl_2_	Si increases the concentration of Fe, Mg, K and Ca.	(Javed et al., 2020)
Pepper	2.0 mM Na_2_SiO_3_	0.1 mM CdCl_2_	Cadmium significantly reduces K and Ca concentration, but silicon promotes the uptake of these two elements, keeping their levels roughly the same as the control (no-stress).	(Kaya et al., 2020)
Wheat	1 mM Na_2_SiO_3_	50, 100 and 200 μM CdCl_2_	N and K concentrations are reduced by cadmium, which is alleviated by silicon.	(Rahman et al., 2021a)
Wheat	3 mM Na_2_SiO_3_	25 mg kg^-1^	Cadmium poisoning reduces Mg^2+^, Zn^2+^, Fe^2+^ in roots and Mg^2+^, Zn^2+^ in the shoots, while silicon application increases Mg^2+^, Zn^2+^, Fe^2+^ in roots and Mg^2+^, Zn^2+^ in the shoots under cadmium poisoning.	(Thind et al., 2020)
Maize	10 µM Na_2_SiO_3_	100 µM CdCl_2_	Cadmium poisoning reduces Ca contents in seedlings, and silicon application increases Ca contents. Cadmium poisoning increases S contents in seedlings, and reduces S contents in seedlings.	(Singh et al., 2019)
Pigeonpea	300 mg kg^−1^ K_2_SiO_3_	25 and 50 mg kg^−1^ CdSO_4_	Cadmium toxicity causes a significant decrease in N, P, K, and Mg content in plant leaves, and the addition of silicon to cadmium-poisoned plants increases N, P, K, and Mg content.	(Garg and Singh, 2018)
Cowpea	1.25 and 2.50 mM Na_2_SiO_3_·9H_2_O	500 µM CdCl_2_	Cadmium reduces the contents of macronutrients (P, K, Ca, Mg and S) and micronutrient (Zn, Cu, Fe, Mn and Mo). However, 500 M cadmium + 2.50 mM silicon treatment significantly increases all of the above elements.	(Pereira TS et al., 2018)
*Gladiolus grandiflora* L.	200 mg L^−1^ Si	50 mg kg^−1^ CdSO_4_·8H_2_O	Cadmium poisoning reduces S, Mn, Ca, Mg, and K contents in roots and shoots and silicon application increased S, Mn, Ca, Mg, and K contents in roots and shoots.	(Zaheer et al., 2018)
Peas	1.8 mM H_4_SiO_4_	20 µM CdSO_4_	Si reduces Cd translocation in shoots through the regulation of Fe transport.	(Rahman et al., 2017)
Rice	2.5 mM Nano-silicon	20 μM Cd^2+^	Nanosilicon increase Mg, Zn, Cu, Mn and root Fe but reduce Ca, Mn, Cu and shoot Fe	(Wang et al., 2015)

Although some studies have been conducted to reveal the effect of silicon on plant mineral elements by silicon under cadmium stress, most of them only demonstrated the results, lacking the exploration of the deep mechanism analysis. Thus, how silicon regulates the uptake of mineral elements and how these mineral elements enhance cadmium resistance in plants remain largely unclear.

## 7 Silicon-induced antioxidant defenses

**Figure 2:**
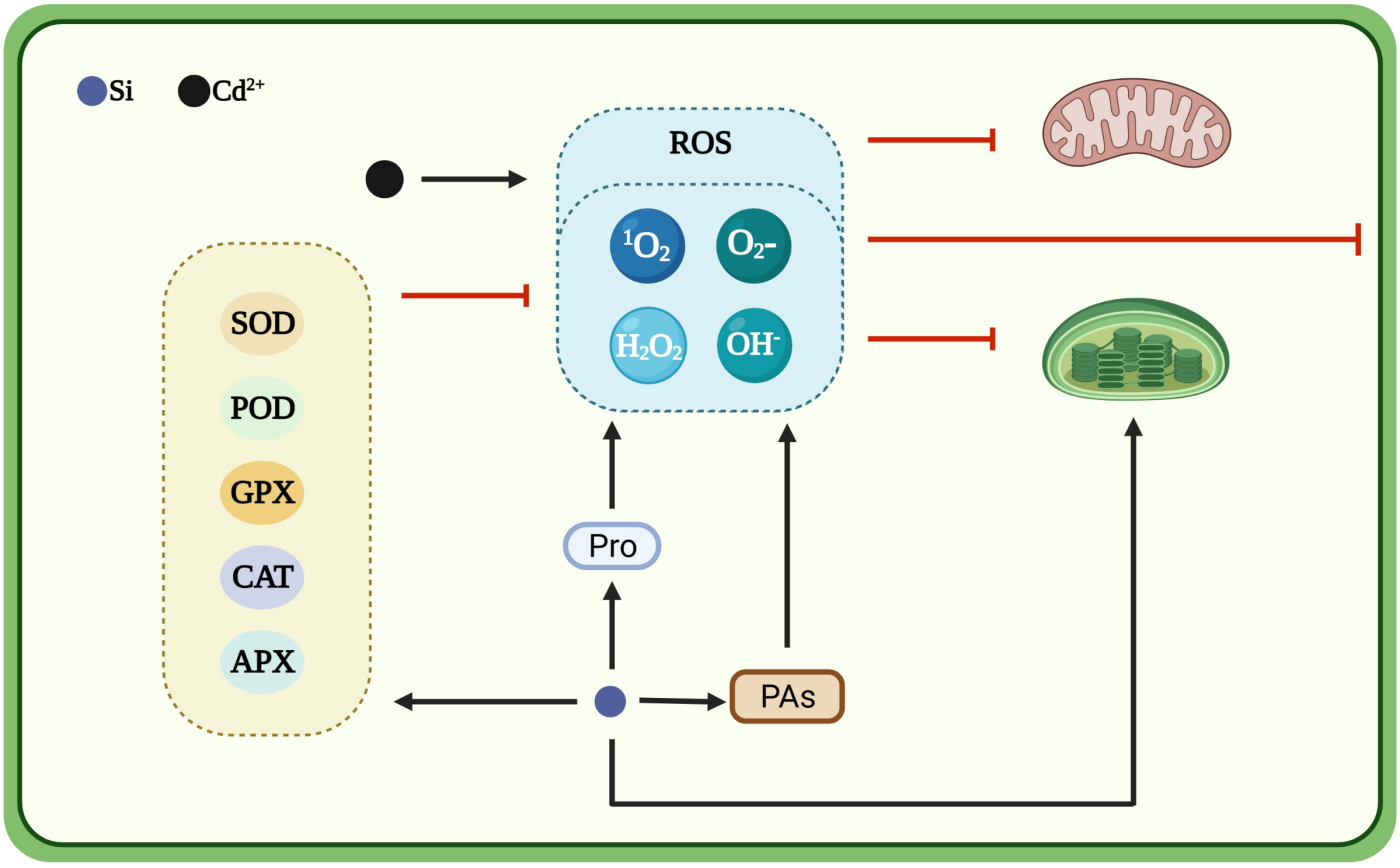
Silicon-induced antioxidant defense system mitigates the toxic effects of cadmium. Cartoon pictures were created with BioRender.com.

The concentration of ROS in plant cells is normally low and poses little danger to cellular functioning. However, upon exposure to heavy metal toxicity, ROS concentrations increase dramatically, triggering a series of physiological and biochemical changes, which eventually cause yield reduction and even plant death. Cadmium increases the production of ROS in plants and causes oxidative stress (Schutzendubel et al., 2001; Farooq et al., 2013). Under cadmium toxicity, large amounts of ROS, including singlet oxygen (^1^O_2_), superoxide ion ( ${\text{O}}_{2}^{-}$ ), hydrogen peroxide (H_2_O_2_) and hydroxyl radical (OH^−^), accumulate in plant cells (Gallego et al., 2012; Liu et al., 2013b). Plants synthesize antioxidant enzymes, such as superoxide dismutase (SOD), peroxidase (POD), catalase (CAT), ascorbate peroxidase (APX) and guaiacol peroxidase (GPX), to cope with the adverse effects of high ROS accumulation under cadmium stress (Li et al., 2018). Silicon can enhance antioxidant capacity of plants (**Figure 2**). Silicon dioxide nanoparticles stimulate the antioxidant defense system in wheat and rice (Tripathi et al., 2017). The application of silicon increases the activities of SOD, POD, CAT, APX and GPX in different wheat cultivars under cadmium toxicity and the degree of enhancement in antioxidant enzyme activity is higher in cadmium-tolerant cultivars than in cadmium-sensitive cultivars (Naeem et al., 2018; Hussain et al., 2019). Foliar spray of silicon on rice plants under cadmium toxicity increases the branch POD and SOD activities and decreases CAT activity (Wang et al., 2015). Similarly, in ginger, silicon increases SOD, POD and CAT activities under cadmium toxicity (Chen et al., 2020). Exogenous silicon treatment increases SOD, CAT and APX activities in cucumber leaves. However, in tomato leaves, silicon increases only SOD activity and decreases other antioxidant enzyme activities under cadmium toxicity (Wu et al., 2015). Moreover, silicon promotes the rapid accumulation of polyamines, scavenges free radicals and prevents heavy metal-induced oxidative damage (Das et al., 2022). Spray application of silicon onto the leaf surface enhances the production of glutamyl kinase (the first committed enzyme in the proline biosynthesis pathway) and decreases the production of proline oxidase (responsible for the denaturation of proline molecules) to promote proline synthesis and help plants to cope with oxidative stress (Elnaz et al., 2021).

By contrast, some studies demonstrated that silicon reduces antioxidant enzyme activity under cadmium stress. For instance, compared to cadmium treatment alone, the presence of silicon reduces the SOD, POD, CAT and APX activity in *Solanum nigrum* L. (Liu et al., 2013b). In wheat, cadmium stress increased SOD, CAT and POD activities by 77.18%, 76.95% and 108.33%, respectively, compared with the control, whereas foliar application of 4.50 mM silicon improved the SOD, POD and CAT activities by 23.91%, 32.01% and 69.76%, respectively (Heile et al., 2021). These contrasting results may be caused by differences in plant species, cultivars, age, silicon source (silicon or nano-silicon), treatment time and experimental conditions (**Table 3**).

**Table 3:** Effect of silicon on plant antioxidant system under cadmium poisoning.

Species	Silicon concentration	Cadmium concentration	Results	Reference
Maize	6 mM K_2_SiO_3_	500 µM CdCl_2_	SOD, APX, POD↑	(Saleem et al., 2022)
Rice	2.5 mM SiNPs	50 μM CdCl_2_·2.5H_2_O	APX, GSH↑	(Riaz et al., 2022)
Pepper	2.0 mM Na_2_SiO_3_	0.1 mM CdCl_2_	GSH, ASA, SOD, POD, CAT↑	(Kaya et al., 2020)
Maize	10 μM Na_2_SiO_3_	100 μM CdCl_2_	SOD, APX, CAT, GR, DHAR, MDHAR, ASA, GSH↑	(Singh et al., 2019)
Rape	0.6 mM Na_2_SiO_3_·9H_2_O	50 μM CdCl_2_·2.5H_2_O	SOD, CAT, POD↑	(Zong et al., 2022)
Basil	1 and 2 mM Na_2_SiO_3_	25 and 50 mg kg^−1^ Cadmium nitrate	SOD, CAT, APX↑; Proline↓	(Gheshlaghpour et al., 2021)
*Isatis cappadocica* Desv	0.5, 1and 2 mM Na_2_SiO_3_	600 μM CdCl_2_	GST, GR↑	(Azam et al., 2021)
Lettuce	1 mM Si solution	1 mM CdCl_2_	SOD, CAT↑; APX↓	(Pereira et al., 2021)
Wheat	3 mM SiNPs	25 mg kg^−1^ CdCl_2_	SOD, POD, APX, CAT↑	(Thind et al., 2021b)
Ajwain	1.5 and 3 mM K_2_SiO_3_	1.5 and 3 mM CdCl_2_	CAT, APX↑	(Javed et al., 2020)
Wheat	1 and 3 mM Na_2_SiO_3_	50 and 200 µM CdCl_2_	CAT, POD, SOD↑	(Rahman et al., 2021b)
Wheat	3 mM Na_2_SiO_3_	25 mg kg^−1^ Cd^2+^	SOD, POD, CAT, APX, ASA, GSH↑	(Thind et al., 2020)
Wheat	1 mM Na_2_SiO_3_	50, 100 and 200 μM CdCl_2_	CAT, SOD, POD, Proline↑	(Rahman et al., 2021b)
Wheat	300, 600, 900 and 1200 mg kg^-1^ SiNPs	7.38 mg kg^-1^ Cd^2+^	SOD, POD↑	(Ali et al., 2019)
Rice	1.5 mM NaSiO_3_·9H_2_O	100 μM CdCl_2_	SOD, POD, CAT↑	(Chen et al., 2019)
Wheat	1 g kg^-1^ Organosilicon and Sodium silicate	2.82 mg kg^-1^ Cd^2+^	POD↑; SOD, CAT, GSH↓	(Huang et al., 2019)
Wheat	300, 600, 900 and 1200 mg L^-1^ SiNPs	7.38 mg kg^-1^ Cd^2+^	SOD, POD, CAT↑	(Hussain et al., 2019)
Wheat	3 mM K_2_SiO_3_.nH_2_O	2 mM Cd^2+^	Proline, AsA, GSH, SOD, CAT, POD↑	(Howladar et al., 2018)
Rice	1, 2, 4 and 6 g kg^−1^ Na_2_SiO_3_·9H_2_O	3.51 mg kg^-1^	SOD, POD, APX, CAT↑	(Li et al., 2018)
*Pfaffia glomerata* (Spreng.)	2.5 mM Na_2_SiO_3_	50 μM CdCl_2_	SOD, POD↑	(Pereira TS et al., 2018)
Cowpea	1.25 and 2.50 mM Na_2_SiO_3_·9H_2_O	500 µM CdCl_2_	SOD, CAT, APX. POX↑	(Pereira AS et al., 2018)
Wheat	1 mM Na_2_SiO_3_·9H_2_O	0, 5 and 20 μM CdCl_2_·3/2H_2_O	SOD, POD↑	(Shi et al., 2018)
Cabbage	5 μM Na_2_SiO_3_	1, 5 μM CdCl_2_	SOD, APX, CAT↑	(Wu et al., 2018)
*Gladiolus grandiflora* L.	200 mg L^−1^ Si	50 mg kg^−1^ CdSO_4_·8H_2_O	SOD, POD, CAT, APX↑	(Zaheer et al., 2018)
*Arabidopsis thaliana*	400 mg kg^−1^ Na_2_SiO_3_	100 mM Cd^2+^	APX, CAT, GR↑	(Carneiro et al., 2017)
*Brassica napus* L	1 mM SiO_2_	0.5 and 1.0 mM CdCl_2_	AsA, GSH, APX, MDHAR, DHAR, GR, CAT, Gly I, Gly II↑	(Hasanuzzaman et al., 2017)
Peas	1.8 mM H_4_O_4_Si	20 µM CdSO_4_	CAT, POD, GR, SOD↑	(Rahman et al., 2017)
Alfalfa	1 mM K_2_SiO_3_	1 mM CdCl_2_	CAT, APX, SOD, Methionine and Proline↑ GR↓	(Kabir et al., 2016)
Rice	200 μM K_2_SiO_3_	2 mM Cd(NO_3_)_2_	CAT↑; SOD, GPX, APX↓	(Srivastava et al., 2015)
Rice	2.5 mM Nano-silicon	20 μM Cd^2+^	GSH, SOD, Shoot POD, Root CAT↑ Root POD, Shoot CAT↓	(Wang et al., 2015)
Tomato Cucumber	0.5 mM (cucumber) and 2 mM (tomato) Na_2_SiO_3_·9H_2_O	100 μM CdCl_2_	SOD, CAT, GR(tomato cucumber), APX(tomato)↑; APX(cucumber)↓	(Wu et al., 2015)
Cotton	1 mM Na_2_SiO_3_	1 and 5 mM CdCl_2_	SOD, GPX, CAT, APX↑	(Farooq et al., 2013)
*Solanum nigrum* L	1 mM Na_2_SiO_3_	100 mM CdCl_2_	SOD, POD, CAT, APX↓	(Liu et al., 2013a)
Maize	0.08 mM Na_2_SiO_3_	5 and 10 μM Cd(NO_3_)_2_·4H_2_O	SOD, POX↑; CAT↓	(Lukačová et al., 2013)
Peanut	1.8 mM Si	200 μM Cd^2+^	SOD, POD, CAT↑	(Shi et al., 2010)

"↑"="increased", "↓"="decreased".

## 8 Silicon adjusts the water balance

Water is the most important constituent of living organisms. Every plant cell requires water for survival. Without water, plants cannot perform photosynthesis (the process of food production), respiration nutrient translocation to different plant parts. Adverse environmental conditions such as drought, salt, evaporation, chilling and heavy metal toxicity can disturb plant water balance, which in turn inhibits plant growth (Bray, 1997; Tchounwou et al., 2012).

Cadmium is mainly present in the soil as the divalent cation (Cd^2+^), which is absorbed by the root system and then translocated to the shoot through the xylem along the transpiration stream (Sterckeman and Thomine, 2020). Because transpiration pull is the main driving force for cadmium transport from the root system to the aboveground parts, lower transpiration rate is likely to reduce cadmium accumulation in the leaf (Uraguchi et al., 2009; Zulfiqar et al., 2021). The application of silicon significantly reduces stomatal conductance and subsequently constrains the transpiration rate, which reduces cadmium transport from roots to shoots in wheat (Naeem et al., 2018). Similarly, silicon reduces stomatal conductance and transpiration rate in *Cannabis sativa* under cadmium stress (Luyckx et al., 2021). In rice, cadmium significantly reduces transpiration, and the reduction in transpiration increases further upon the addition of silicon. Interestingly, the transpiration rate of plants treated with 0.2 mM silicon is significantly lower than that of plants treated with 0.6 mM silicon (Nwugo and Huerta, 2008). Rizwan et al. (2016) indicated that silicon-induced reduction in transpiration would reduce cadmium translocation to shoots. Additionally, silicon application decreases transpiration rate by 65% and 42% in - cadmium and + cadmium plants, respectively (Nwugo and Huerta, 2011).

Contrary to the above result, there are accumulating experiments proved that under cadmium stress silicon enhances gas exchange indices (especially represented by transpiration rate and stomatal conductance) in rice (**Table 4**; Gao et al., 2018; Li et al., 2018; Rehman et al., 2019; Rizwan et al., 2019; Sohail et al., 2019; Huang et al., 2021; Mapodzeke et al., 2021), wheat (Alzahrani et al., 2018; Shi et al., 2018; Ali et al., 2019; Hussain et al., 2019), peas (Jan et al., 2018), beans (Rady et al., 2019; Ahmad et al., 2021; El-Saadony et al., 2021), maize (Sohail et al., 2019), gladiolus (Zaheer et al., 2018), pepper (Kaya et al., 2020), ajwain (Javed et al., 2020), cabbage (Yang et al., 2018). In comparison with control (non-stressed) conditions, cadmium stress conditions reduce photosynthetic rate (37.29%), transpiration rate (37.28%), stomatal conductance (38.09%) and chlorophyll content (14.13%). Silicon supplementation improves plant tolerance to cadmium stress. The greatest influence on all physio-biochemical attributes was noticed in plants supplemented with 4.50 mM silicon under cadmium stress. In the presence of 4.50 mM silicon, the most promising level, increases in the photosynthetic rate (45.77%), rate of transpiration (38.60%), stomatal conductance (42.85%), chlorophyll contents (45.77%) and water use efficiency (7.77%) compared with the relevant control (Heile et al., 2021). In rice, foliar spray of silicon reduces the accumulation of cadmium in leaves but increases the transpiration rate and stomatal conductance of leaves, compared with the control, under cadmium stress. Furthermore, structural equation modeling indicated that transpiration rate and stomatal conductance have negative effects on cadmium concentration in rice (Gao et al., 2018). Compared with the control (no-stress condition), cadmium stress reduces the transpiration rate and stomatal conductance of wheat leaves, but external silicon application significantly alleviates the inhibitory effect of cadmium on these indicators and subsequently enhances the water use efficiency (WUE) of plants (Howladar et al., 2018). Notably, in this study, relative leaf water content (RWC), a common indicator of leaf water balance, was also enhanced by silicon under stress (Howladar et al., 2018). Similar results have also been reported in bean (Rady et al., 2019; Ahmad et al., 2021; El-Saadony et al., 2021), pea (Jan et al., 2018) and pepper (Kaya et al., 2020). Silicon addition maintains RWC in wheat cells and tissues under cadmium poisoning (Alzahrani et al., 2018; Heile et al., 2021). The addition of silicon significantly increases RWC to maintain cell expansion pressure and protects plants from wilting and cell relaxation to mitigate plant cadmium toxicity (Rahman et al., 2021a). In addition, and more importantly, an increase in the leaf water content by silicon could dilute the cadmium concentration, subsequently reduce cadmium toxicity in plants (Kaya et al., 2020).

**Table 4:** Effect of silicon on the water balance under cadmium poisoning.

Species	Silicon concentration	Cadmium concentration	Effect of silicon on, Tr, Gs, RWC and WUE	Reference
Peas	0.5, 1 and 1.5 mM Na_2_SiO_3_ and K_2_SiO_3_	20 mg kg^−1^ CdCl_2_	Tr, Gs, RWC↑	(Batool et al., 2022)
Wheat	1 mM Na_2_SiO_3_	100 and 200 μM CdSO_4_·8H_2_O	RWC↑	(Saber et al., 2022)
Beans	2 mM Na_2_SiO_3_	75 mg kg^-1^ Cd^2+^	Tr, Gs, RWC↑	(Ahmad et al., 2021)
Summer savory	0.75, 1.5 and 2.25 mM SiNPs	10 and 20 mg kg^−1^ CdCl_2_	RWC↑	(Elnaz et al., 2021)
Beans	2.5, 5 mM	18.6 mg kg^−1^ Cd^2+^	Tr, Gs, RWC↑	(El-Saadony et al., 2021)
Wheat	1.5, 3.0 and 4.5 mM silicon compounds (K_2_SiO_3_ and CaSiO_3_)	20 mg kg^−1^ CdCl_2_	Tr, Gs, RWC↑	(Heile et al., 2021)
Rice	1.5 mM Na_2_SiO_3_·9H_2_O	100 μM CdCl_2_·2H_2_O	Tr, Gs↑	(Huang et al., 2021)
Hemp	2 mM H_2_SiO_3_	20 μM CdCl_2_	Tr, Gs↓	(Luyckx et al., 2021)
Rice	5, 15 μM Na_2_SiO_3_·9H2O	15 μM CdCl_2_	Tr↑	(Mapodzeke et al., 2021)
Wheat	1, 3 mM Na_2_SiO_3_	50, 200 μM CdCl_2_	RWC↑	(Rahman et al., 2021a)
Ajwain	1.5, 3 mM K_2_SiO_3_	1.5, 3 mM CdCl_2_	Tr, Gs↑	(Javed et al., 2020)
Pepper	2 mM Na_2_SiO_3_	0.1 mM CdCl_2_	RWC↑	(Kaya et al., 2020)
Wheat	300, 600, 900 and 1200 mg kg^-1^ SiNPs	7.38 mg kg^−1^ Cd^2+^	Tr, Gs↑	(Ali et al., 2019)
Sunflower	100 mg kg^-1^ Na_2_SiO_3_	20, 40 mg kg^−1^ CdSO_4_	RWC↑	(Ashraf et al., 2019)
Wheat	300, 600, 900 and 1200 mg L^-1^ SiNPs	7.38 mg kg^−1^ Cd^2+^	Tr, Gs↑	(Hussain et al., 2019)
Beans	6 mM K_2_SiO_3_·nH_2_O	1.5 mM CdCl_2_	Tr, RWC↑	(Rady et al., 2019)
Rice	0.03% w/w K_2_SiO_3_	0.52 mg kg^-1^ CdSO_4_·8/3H_2_O	Tr, Gs↑	(Rehman et al., 2019)
Rice	5, 10, 15 and 20 mg L^-1^ Nanosilica	7.86 mg kg^−1^ Cd^2+^	Tr, Gs↑	(Rizwan et al., 2019)
Rice, Maize	150 mg kg^-1^ SiO_2_	6.81 mg kg^-1^ Cd^2+^	Tr, Gs↑	(Sohail et al., 2019)
Wheat	2, 4 and 6 mM K_2_SiO_3_·nH_2_O	2 mM Cd^2+^	Tr, Gs, RWC↑	(Alzahrani et al., 2018)
Rice	2.5 mM Si	0.3 mg kg^-1^ Cd^2+^	Tr, Gs↓	(Gao et al., 2018)
Wheat	3 mM K_2_SiO_3_·nH_2_O	2 mM Cd^2+^	Tr, Gs, RWC, WUE↑	(Howladar et al., 2018)
Beans	2 mM Na_2_SiO_3_	150 mg L^-1^ CdSO_4_·8H_2_O	Tr, Gs, RWC↑	(Jan et al., 2018)
Rice	1, 2, 4 and 6 g kg^−1^ Na_2_SiO_3_·9H_2_O	3.51 mg kg^-1^ Cd^2+^	Tr↑	(Li et al., 2018)
Wheat	0.6 mM SiO_2_·nH_2_O	15 mM CdCl_2_·H_2_O	Tr, Gs↓	(Naeem et al., 2018)
Wheat	1 mM Na_2_SiO_3_·9H_2_O	5, 20 μM CdCl_2_·3/2H_2_O	Tr, Gs↑	(Shi et al., 2018)
Cabbage	1.2 mM Na_2_SiO_3_·9H_2_O	50 μM Cd^2+^	Tr, Gs↑	(Yang et al., 2018)
Gladiolus	200 mg L^-1^ Si	50 mg kg^-1^ CdSO_4_·8H_2_O	Tr, Gs↑	(Zaheer et al., 2018)
Cotton	1 mM Na_2_SiO_3_	1 and 5 mM CdCl_2_	Tr, Gs, WUE↑	(Farooq et al., 2013)

"↑"="increased", "↓"="decreased".

Plant leaf water content depends on the dynamic balance between water loss from leaves and water uptake by roots; thus, changes in both these processes affect the plant water status. Transpiration is the primary mode of water loss from shoots (Bernacchi and Vanloocke, 2015; Wang et al., 2018). As mentioned above, silicon-induced decrease in transpiration not only reduces the translocation of cadmium from roots to leaves but also reduces leaf water loss, resulting in higher RWC than the cadmium treatment (Chen et al., 2018).

In addition to water loss, water uptake also influences the RWC (Steudle, 2000; Liu et al., 2014). The RWC of wheat significantly decreases under drought, salinity and cadmium stresses. However, the presence of silicon significantly reduces the destructive effects of the above stresses on RWC. According to these data, we can infer that the mitigation effect of silicon on RWC is similar in the three stress conditions. In other words, the effect of silicon on leaf water content is independent of the stress type (Alzahrani et al., 2018). Therefore, we can hypothesize that the mechanism of silicon on enhancing water balance revealed in other stresses may also apply to cadmium toxicity. Liu et al. (2014) revealed that silicon application maintains the water balance in sorghum by elevating root water uptake under stress conditions. This phenomenon has been confirmed in other studies (Liu et al., 2015; Zhu et al., 2015; Chen et al., 2016).

**Figure 3:**
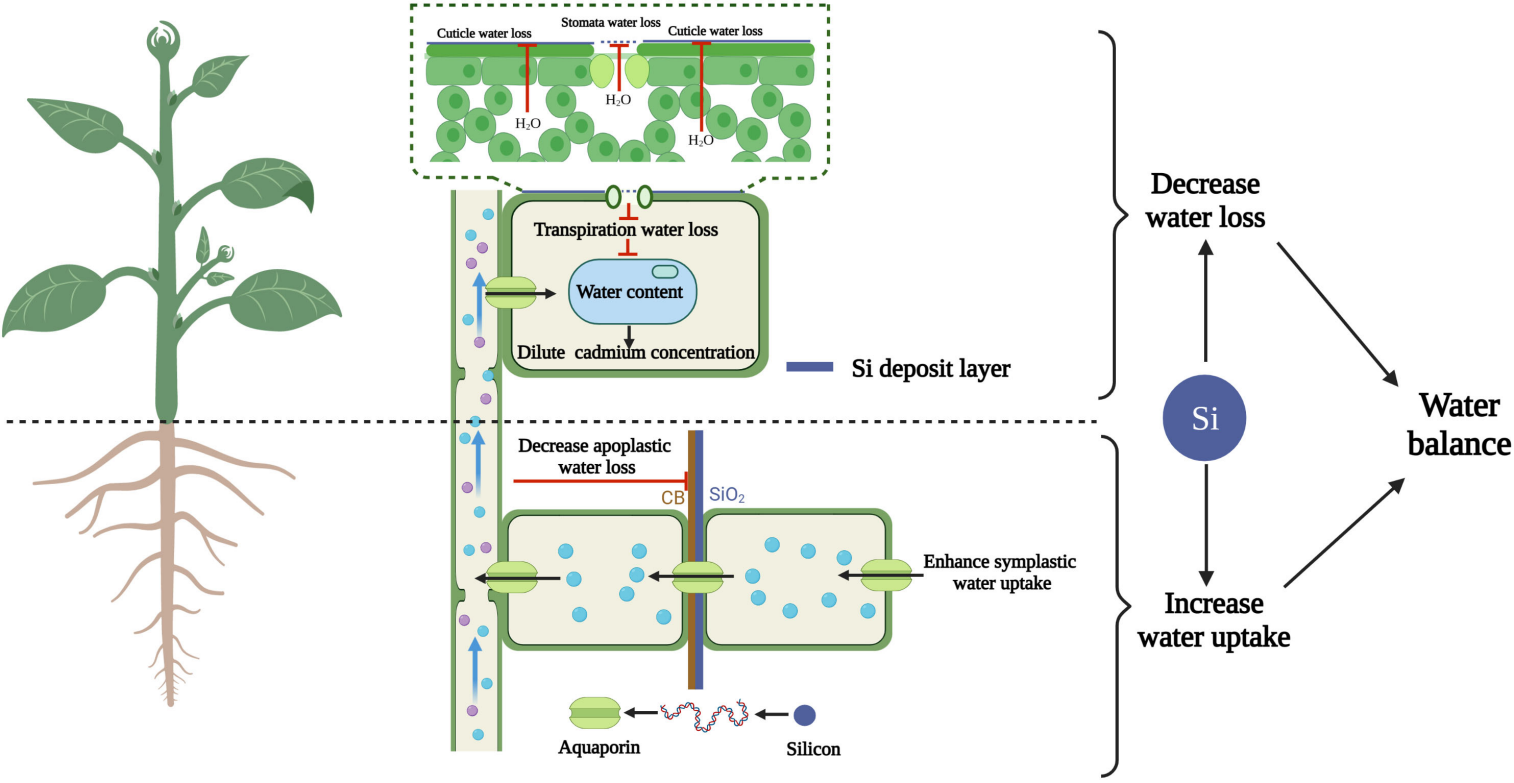
Silicon-diluted cadmium concentration by maintaining the water balance (increasing root water uptake and decreasing leaf water loss). Cartoon pictures were created with BioRender.com.

Water channel proteins play a major role in regulating root water uptake and shoot water transport under stress (Hachez et al., 2012). Silicon-mediated increase in the expression of the plasma membrane intrinsic protein water channel protein (*PIP*) gene is associated with an increase in root hydraulic conductivity and water uptake (Rios et al., 2017). An effect of silicon on aquaporin gene expression has been observed in sorghum (Liu et al., 2014), cucumber (Zhu et al., 2015) and barley (Celikkol and Erkan, 2016). In tobacco, we recently observed that the expression of *NtPIP* genes was strongly enhanced under cadmium stress (unpublished data). In addition to the expression of aquaporin genes, the content of aquaporin proteins is also influenced by silicon. In rapeseed plants under drought stress, silicon application promotes the expression of *BnPIP1*, *BnPIP2-1-7* and *BnTIP1;1* and the accumulation of aquaporins (Gao et al., 2006; Saja-Garbarz et al., 2022). These findings show that water channel protein transport activity can be regulated at the post-transcriptional level. The effect of silicon on plant water balance under cadmium stress is summarized in **Figure 3**.

## 9 Conclusions and perspectives

There is increasing evidence that silicon can improve plant growth under exposure to cadmium toxicity. The reported mechanisms include complexation and coprecipitation of silicon with cadmium in different plant organs, compartmentalization of cadmium in different subcellular organelles, structural alteration of the plant, reduction of cadmium content, regulation of cadmium transporter gene expression, improvement of plant mineral nutrient supply (**Figure 1**), enhancement of ROS scavenging (**Figure 2**) and maintenance of leaf water balance (**Figure 3**).

However, the molecular mechanism underlying the effect of silicon on plant reactions remains unknown. Further research is needed to determine whether silicon directly or indirectly participates in plant physiological responses or gene expression regulation under cadmium stress. An increasing number of studies show that the function of silicon is mediated by signaling messengers, such as plant hormones, ROS and Ca^2+^. Thus, the crosstalk between silicon and signaling messengers may constitute an important research focus to elucidate the mechanism of silicon-mediated increase in cadmium tolerance in plants.

## Author contributions

LH: Writing-original draft; SJ: Visualization, Writing-original draft; YZ: Writing-review & editing; XW: Methodology; LZ: Writing-review & editing; PL: Conceptualization, Writing review & editing, Funding acquisition. All authors contributed to the article and approved the submitted version.
